# Weight-Teasing and Eating Disorders—A Comparative Study in Adolescent and Adult Samples

**DOI:** 10.3390/children9111655

**Published:** 2022-10-29

**Authors:** Laura O. Gallardo, J. Javier Plumed-Domingo, Luis Rojo-Moreno

**Affiliations:** 1 Department of Psychology and Sociology, Universidad de Zaragoza, 44003 Teruel, Spain; 2 Department of Medicine, Universidad de Valencia, 46010 Valencia, Spain

**Keywords:** adolescents, adults, weight-teasing, eating disorders

## Abstract

Disordered eating, unhealthy weight-control behaviours and body dissatisfaction are associated with diminished mental health. A key aspect that has been identified for the development of disordered eating behaviours and body dissatisfaction is to be teased. Additionally, the literature suggests that weight may affect the relationship between weight-teasing and disordered eating and body dissatisfaction, although this pattern is unclear. This study presents two cross-sectional studies with an adult and an adolescent sample. The adolescent sample comprised 15,224 participants, and the adult sample comprised 321 participants, all from Spain. Hierarchical regression analyses were conducted. Aims: to assess the relations among disordered eating, body dissatisfaction, weight, and weight-teasing among adolescents and adults; and to examine whether weight-teasing, weight, age, and gender predict disordered eating and body dissatisfaction in adults and adolescents. The results showed that adolescent girls presented greater effects, and all interactions were significant between weight-teasing and eating disorders. Adults also showed greater effects for women, and only eating disorder interactions were significant. Weight-control behaviours did not show any relationship with weight-teasing. Conclusion: Different approaches may be used in the prevention of eating disorders. Our results show that weight-teasing affects adults differently from adolescents.

## 1. Introduction

Due to the physical, maturational and psychological changes that occur during this period, adolescence is a risk period for developing eating disorders [1]. Evidence indicates that disordered eating, unhealthy weight–control behaviours and body dissatisfaction are related [2] and associated with diminished mental health in children, adolescents and adults (see review [3]).

An aspect identified as particularly relevant for developing disordered eating behaviours and body dissatisfaction is being teased [4,5]. Teasing has been defined as an agent’s personal communication directed toward another person, which includes three components: aggression, humour and ambiguity [6]. Research has associated being weight-teased as a child, adolescent, or adult of both genders with body dissatisfaction and dietary restraint and diminished mental health in different developmental stages (e.g., [3,7,8,9]). In addition, being teased about one’s weight has been positively associated with unhealthy weight-control behaviours, dysfunctional eating cognitions, bulimic behaviours, depressive symptoms, and low self-esteem [1,2,4,10]. Likewise, being teased about weight and size while growing up has been related to body dissatisfaction and evaluation of one’s appearance in adulthood [5]. 

Teasing about one’s weight implies that weight may be involved in the relationship between weight-teasing and the development of disordered eating behaviours, as Neumark-Sztainer et al. [2] reported. Nevertheless, regarding the subject’s weight pattern, the results are inconsistent. Specifically, researchers have indicated that weight-teasing was a significant predictor of body dissatisfaction, dietary restraint, self-esteem, and depression even after controlling for body mass index (BMI) in adolescents [1,3,11]. Concerning age, a study with an older sample showed that BMI was related to youth and adult teasing. However, after controlling for BMI, only adult teasing predicted body dissatisfaction in overweight and obese women [12]. In contrast, despite the positive association between weight-teasing and body dissatisfaction, [13] did not find any association when controlling for BMI, self-esteem, depression and other control variables in adolescent girls. Taken together, these results suggest a more complex relationship between weight-teasing, disordered eating behaviours and body dissatisfaction.

Due to these discordant results, more elaborated relationships have been examined. Several studies have assessed how weight-teasing mediates the correlation between BMI and body dissatisfaction, although with inconsistent results. A study with an adolescent sample of females from the USA found the relationship between BMI and body dissatisfaction was fully mediated by weight-teasing [14]. In contrast, Lunner et al. [15] replicated the earlier study, finding a partially mediated model in only three samples of female adolescents from Sweden and Australia. Research has also assessed the possibility of an interaction between weight-teasing and weight. Bardone-Cone et al. [16] found a significant interaction between weight-teasing and BMI in the prediction of weight concerns in young adults. In contrast, Hayden-Wade et al. [17], in a sample of early adolescents, found that weight-teasing and BMI predicted weight concerns, but their interaction did not reach statistical significance. Likewise, Eisenberg et al., [10] found that the interaction between weight-teasing and weight status did not show clear patterns and did not predict body dissatisfaction, self-esteem or depressive symptoms in adolescents. A study developed in Italy by Guardabassi et al. [18] found that weight-teasing mediates the relation between BMI and health-related quality of life in middle childhood. Nevertheless, findings provide strong support for an interactive effect of weight and weight-teasing for the development of body image disturbance, as reported by Thompson et al. [14].

In summary, past research suggests that weight may affect the relationship between weight-teasing and disordered eating and body dissatisfaction, although this pattern is unclear. Thus, we conducted two cross-sectional studies with an adult and an adolescent sample. Specifically, the aims of the present studies were: (a) to assess the relations among disordered eating, body dissatisfaction, weight and weight-teasing among adolescents and adults; and (b) to examine whether weight-teasing, weight, age, and gender predict disordered eating and body dissatisfaction in adolescents and adults. In addition, the interaction between weight-teasing and weight (moderator effect) was examined in both samples.

## 2. Study 1

### 2.1. Materials and Methods

#### 2.1.1. Participants

Participants were 15,224 secondary school students from the region of Valencia (Spain). The sample comprised 49.7% females and 50.3% males. Adolescents’ ages ranged from 12 to 18 years (*M* = 15.25, *SD* = 1.20). Participants were distributed in the four grades of Compulsory Secondary Education (CSE) of the Spanish school system (7th to 10th grade in the USA). The total sample obtained a mean BMI of 21.73 kg/m^2^ (*SD* = 3.93 kg/m^2^).

#### 2.1.2. Measures

Perception of Weight-Related Teasing Subscale (POTS-WT; [14]). The POTS-WT measures an individual’s history of being teased about weight. It consists of 6 items to assess both the frequency of teasing (ranging from never = 1 to very often = 5) and its impact on the individual, rated on a Likert scale ranging from 1 (not upset) to 5 (very upset). Example items are: “People made fun of you because you were heavy” or “People pointed at you because you were overweight”. Higher scores denote greater weight-teasing exposure and sensitivity. The validated Spanish version [19] has an internal consistency of 0.95 (Cronbach’s alpha in our sample was 0.94). 

Body Shape Questionnaire (BSQ; [20]). The BSQ assesses the degree of an individual’s dissatisfaction with their physical appearance with items like: “Have you ever felt so bad about your shape that you have cried?” or “Has being naked, such as when taking a bath, made you feel fat?” The scale includes 34 items related to self-image rated on a Likert scale ranging from 1 (never) to 6 (always). The Spanish version, validated by Raich Escursell et al. [21], has an internal consistency of 0.93 to 0.97 (Cronbach’s alpha in our sample was 0.97). 

Eating Disorder Inventory-2 Drive for Thinness (EDI-2 DT; [22]). The EDI-2 DT subscale includes 7 items related to the desire to lose weight, dieting and fear of weight gain. Example items are: “I am preoccupied with the desire to be thinner” or “I am terrified of gaining weight.” Participants’ options were rated on a Likert-type scale with six forced-response choices, where never, rarely, or sometimes scores are equal to 0, often is 1, very often is 2, and always is 3. The validated Spanish version [23] has an internal consistency of 0.75 to 0.88 (Cronbach’s alpha in our sample was 0.81).

Eating Disorder Inventory-2 Body Dissatisfaction (EDI-2 BD; [22]). The EDI-2 BD subscale assesses satisfaction with one’s general shape and with particular body parts with items such as: “I feel satisfied with the shape of my body” or “I think my buttocks are too large”. It consists of 9 items concerning body parts or the entire body rated on a 6-point Likert scale where never, rarely, or sometimes scores are equal to 0, often is 1, very often is 2, and always is 3. The Spanish version validated by Garner [23] has an internal consistency of 0.71 to 0.87 (Cronbach’s alpha in our sample was 0.71).

Children’s Eating Attitudes Test (ChEAT: [24]). ChEAT was used to detect disordered eating. It consists of 26 items related to maladaptive or problematic eating attitudes and behaviours among children and adolescents, rated on a 6-point Likert scale where never, rarely, or sometimes scores are equal to 0, often is 1, very often is 2, and always is 3. Example items are: “I am scared about being overweight” or “I feel very guilty after eating”. The internal consistency of the Spanish version [25] was 0.73 (Cronbach’s alpha in our sample was 0.77).

Residual Weight. This was measured by standardised residual scores derived by regressing sex, age, height, height^2^, and Sex × Age onto expected weight. It is measure on a continuum ranging from overweight (positive values) to underweight (negative values). Values around the mean denote unbiased weight. 

#### 2.1.3. Procedure

Before the research was conducted, the study was approved by the health authority’s Department of Public Health Office of the Regional Valencian Government (Spain) (2016/0141), and then by the Board of each school. All schools of the region of Valencia were asked to participate. The participating schools obtained passive consent from participants’ parents or guardians. Adolescents could agree to participate only if their parents or guardians did not return their refusal for their participation. 

Data were collected during regular classroom sessions. Adolescents’ height and weight were measured in situ by specially trained personnel with the same equipment for all participants; the rest of the questionnaire used was administered by teachers. The inclusion criteria were to answer all the questions of the study.

#### 2.1.4. Statistical Analysis

Statistical analysis was performed using the statistical package IBM SPSS Statistics for Windows, Version 25.0. Descriptive statistics of the variables were calculated, as were Pearson’s correlation coefficients assessing the relationships among disordered eating, body dissatisfaction, weight, and weight-teasing in adolescents. A series of hierarchical regression analyses was conducted to determine how weight-teasing, weight, age and gender predict different disordered eating behaviours related to eating attitudes and body dissatisfaction. Specifically, BSQ, EDI-2 DT, EDI-2 BD and ChEAT were predicted by POTS-WT, residual weight, age, and gender. Additionally, the interaction term Residual Weight × POTS-WT was included in Step 2 to examine whether weight moderates the relationship between weight-teasing and disordered eating and body dissatisfaction. When the interaction was non-significant, we concluded that the association between the dependent variable and residual weight did not vary as a function of weight-teasing. When the interaction was statistically significant, weight-teasing was considered to modify the association between weight and the dependent variable, so stratified analyses according to different levels of weight-teasing were performed. The confidence level was 95%. To better understand these effects, interactions were analysed and plotted separately, conducting simple slope analyses at values of one standard deviation above (high) and below (low) the means of the implicated variables [26].

### 2.2. Results of study 1

Descriptive statistics for the study variables and correlations are provided in Table 1. As expected, girls scored moderately higher in BSQ, EDI-2 DT, EDI-2 BD and ChEAT and slightly higher in POTS-WT than boys. Residual weight was positively and moderately related to all the criterion variables and also to POTS-WT scores. Dependent variables were positively and moderately related to POTS-WT. 

Table 2 presents the results of the hierarchical regression analyses. At Step 1, gender, residual weight, and POTS-WT consistently accounted for a significant amount of variance in BSQ, EDI-2 DT, EDI-2 BD, and ChEAT. The regression analysis predicted girls’ higher levels in all the dependent variables than in boys. An independent, small, and positive effect of age remained only when considering BSQ and EDI-2 BD. The set of predictors accounted for 36% of the variance in BSQ, 23% of the variance in EDI-2 DT, 25% of the variance in EDI-2 DT, and 13% of the variance in ChEAT. 

**Figure 1:**
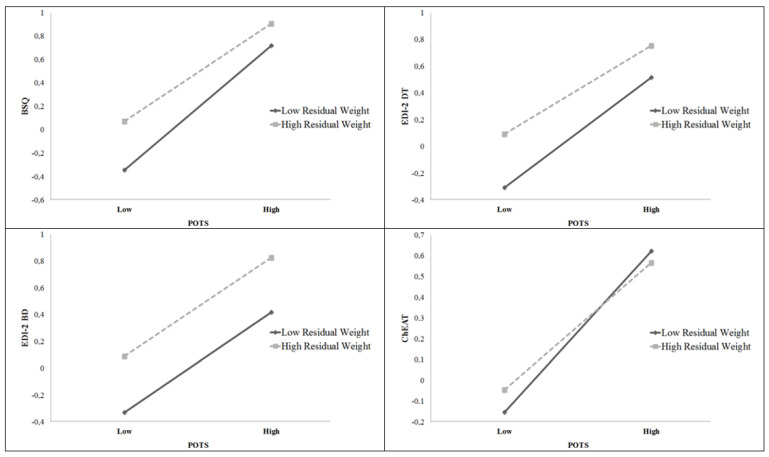
Interaction between residual weight and POTS-WT in predicting BSQ, EDI-2 DT, EDI-2 BD and ChEAT in adolescents.

Significant moderator findings entered in Step 2 are reported in Table 2. Residual weight and POTS-WT interacted to predict BSQ (*t* = −8.82, *p* < 0.001, Δ*R*^2^ = 0.003), EDI-2 DT (*t* = −5.73, *p* < 0.001, Δ*R*^2^ = 0.002), and ChEAT (*t* = −5.40, *p* < 0.001, Δ*R*^2^ = 0.002). This indicates that increments in adolescents’ teasing reduce the slope that relates weight and disordered eating behaviours and attitudes (such as BSQ, EDI-2 DT and ChEAT). The interaction term between residual weight and POTS-WT did not emerge as a significant predictor of EDI-2 BD. The nature of these interactions between residual weight and POTS-WT and their association with adolescents’ disordered eating behaviours and attitudes are illustrated in Figure 1. 

## 3. Study 2

### 3.1. Materials and Methods

#### 3.1.1. Participants

The sample consisted of 321 adults who completed the entire questionnaire used in the present study through the internet. Participants’ ages ranged from 18 to 65 years (*M* = 29.98, *SD* = 11.00). The sample comprised 73.5% females and 26.5% males. The sample distribution by educational level was: 6.23% had finished secondary school, 19.0% had finished high school, 38.32% were studying at university, and 36.45% had finished university. Most of the sample (96.88%) resided in Spain versus 3.12% in other countries. The total sample obtained a mean BMI of 23.16 kg/m^2^ (*SD* = 3.78 kg/m^2^). Participants voluntarily answered an advertisement published on social networks for 2 weeks. 

#### 3.1.2. Measures

Perception of Teasing Scale (POTS-W; [14]). The POTS-WT assesses an individual’s history of being teased about weight and abilities/competencies. It includes 11 items rated on a 5-point Likert scale ranging from 1 (never) to 5 (very often), which measures the frequency of teasing. In addition, each item was followed by a question about the extent to which the experience was upsetting, ranging from 1 (not upset) to 5 (very upset), measuring its impact on the individual. An example item is: “People made jokes about you being too heavy” and “How upset were you?” The POTS-WT score is obtained by adding all the items, with higher scores denoting greater teasing exposure and sensitivity. The validated Spanish version by López-Gimerá et al. [27] POTS-S-WT has an internal consistency of 0.85 (Cronbach’s alpha in our sample was 0.96). 

Weight Control Behaviours. (WCB; [2]). The WCB assesses several weight-related behaviours. Participants were asked to complete the questionnaire indicating whether or not they had engaged in each weight-control behaviour in the last year with the aim of weight loss or weight maintenance. It includes 16 items rated on a 5-point Likert scale, rated as never = 1, 1 to 4 times = 2, 5 to 10 times = 3, more than 10 times = 4, or I am always dieting = 5”. An item example is “How often have you gone on a diet during the last year? By ‘diet’, we mean changing the way you eat so you can lose weight”. Internal consistency from previous research ranged from α = 0.85 to α = 0.70 (α = 0.82 in the present study).

Eating Disorder Inventory-2 Drive for Thinness (EDI-3 DT; [28]) The subscale EDI-3 DT includes 7 items about dieting, preoccupation with diet restrictions, and fear of weight gain. Item examples are: “I think about dieting” or “I feel guilty after overeating.” Participants’ response options ranged from 1 (always) to 6 (never). The validated Spanish version [29] has an internal consistency of 0.92 (Cronbach’s alpha in our sample was 0.90).

Eating Disorder Inventory-2 Body Dissatisfaction (EDI-3 B; [28]). The subscale EDI-3 BD assesses overall feelings of displeasure about the shape and size of one’s overall body and/or specific parts with items such as “I feel fat” or “I think that my thighs are too large”. It consists of 9 items rated on a 6-point Likert scale ranging from 1 (always) to 6 (never). The Spanish version validated by Elosua et al. [29] has an internal consistency of 0.92 (Cronbach’s alpha in our sample was 0.89).

BMI. Height and weight were self-reported by participants. BMI was subsequently computed using the formula: weight (kg)/ height (m^2^). Past research has confirmed the reliability of self-reported weight and height and has considered both weight and height as valid measures (e.g., [30]).

#### 3.1.3. Procedure

The questionnaire used in the present study was retrieved from different social network profiles on the Internet (such as Facebook and Twitter) during April 2013. Before completing the questionnaire, complete information about the project was provided, and participants’ consent was required. Only adults (between 18 and 65 years) were considered participants. 

#### 3.1.4. Statistical Analysis

Descriptive statistics and Pearson’s correlation coefficients were calculated, assessing the relationship among disordered eating, body dissatisfaction, weight, and weight-teasing in adults. Subsequently, a series of hierarchical regression analyses were conducted to determine how weight-teasing, weight, age and gender predicted disordered eating behaviours related to eating attitudes and satisfaction. The dependent variables assessed were WCB, EDI-DT and EDI-BD. The predictors included in the regression analyses were: age, gender, POTS-S-WT and BMI. Furthermore, the interaction between POTS-S-WT and BMI was included in Step 2, examining whether weight moderates the relationship between weight-teasing, disordered eating and body dissatisfaction. A non-significant interaction indicated that the association between the dependent variable and BMI did not vary as a function of weight-teasing. In contrast, when the interaction was statistically significant, weight-teasing modified the association between BMI and the dependent variable. In that case, we stratified the analyses by different levels of weight-teasing and plotted the interactions separately with the same procedure described in Study 1. Statistical analysis was performed using the statistical package IBM SPSS Statistics for Windows, Version 25.0.

### 3.2. Results of Study 2

Table 3 includes the descriptive statistics and correlations of the study variables. Not surprisingly, women scored moderately higher on WCB, EDI-DT and EDI-BD, and slightly higher on POTS-WT than men. Furthermore, men showed higher BMI than women, and BMI was also higher for older adults. As expected, weight and weight-teasing were positively and moderately related to disordered eating behaviours and body dissatisfaction. Moreover, weight and weight-teasing were positive and moderately related. Regarding age, younger adults reported more weight-control behaviours, body dissatisfaction, and disordered eating behaviours.

Table 4 presents the results of the hierarchical regression analyses. At Step 1, gender, age and BMI consistently accounted for a significant amount of variance in the WCB, EDI-DT and EDI-BD. The regression analysis predicted higher levels for women than for men in all the dependent variables, and significantly higher levels of weight-control behaviours, body dissatisfaction, and disordered eating behaviours for younger adults. An independent effect of POTS-S-WT remained only when considering EDI-DT and EDI-BD. The set of predictors accounted for 21% of the variance in WCB, 36% of the variance in EDI-DT, and 42% of the variance in EDI-BD. 

**Figure 2:**
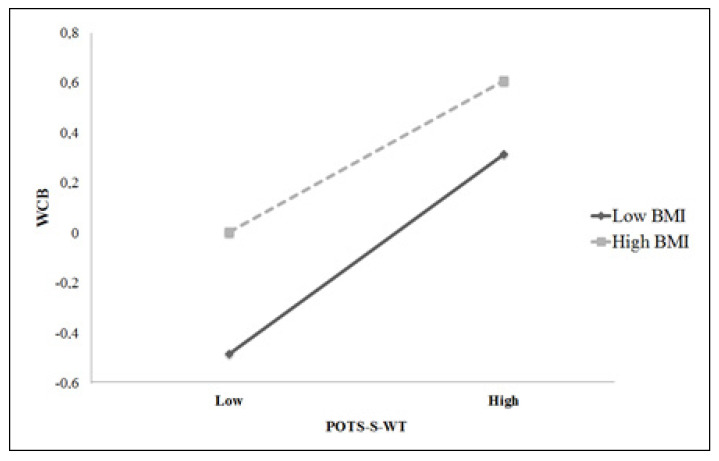

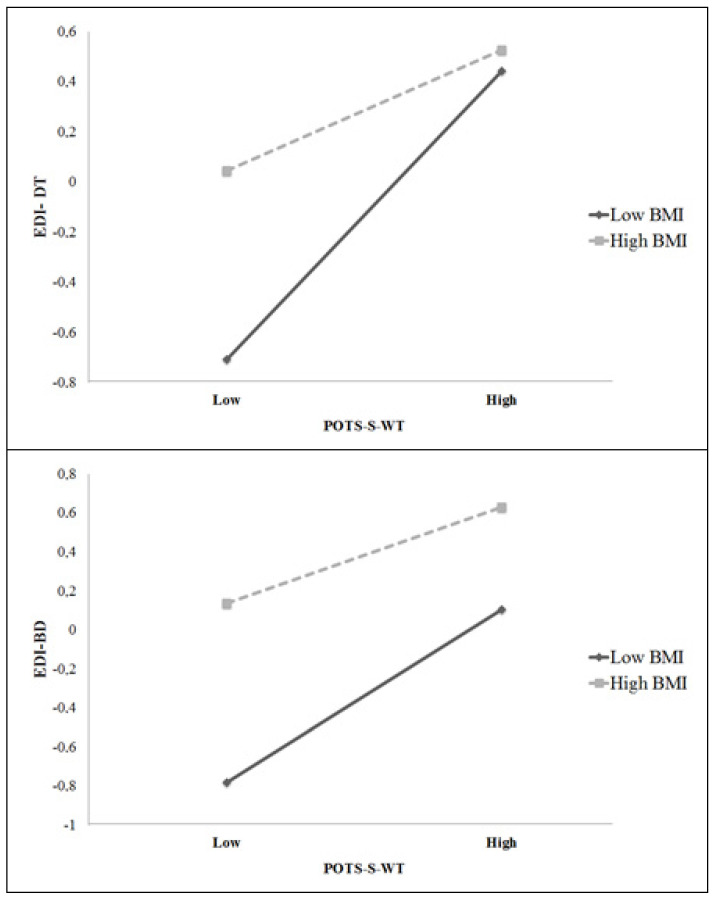
Interaction between BMI and POTS-S-WT in predicting WCB, EDI-DT, and EDI-BD in adults.

Significant moderator findings entered in Step 2 are reported in Table 4. The BMI × POTS-S-WT interaction predicted EDI-DT (*t* = −3.62, *p* < 0.001, Δ*R*^2^ = 0.027) and EDI-BD (*t* = 8.54, *p* < 0.001, Δ*R*^2^ = 0.009). This indicates that changes in individuals’ BMI have a higher impact on the predicted EDI-BD and EDI-DT scores for low weight-teasing than for high weight-teasing. The interaction term between BMI and POTS-S-WT did not contribute to the variance in WCB. These interactions between weight and weight-teasing and their associations with adults’ weight-control behaviours, body dissatisfaction and disordered eating behaviours are illustrated in Figure 2.

## 4. Discussion

The current studies analysed the associations between weight and being weight-teased with body dissatisfaction and disordered eating behaviours in two samples of adolescents and adults. Our results showed a moderation effect of weight when the relationships of weight-teasing and body dissatisfaction or disordered eating behaviours are considered in adolescents and adults. Indeed, when the levels of weight-teasing received are low, greater weight predicts higher levels of disordered eating behaviours and body dissatisfaction. However, when the level of weight-teasing received is high, lower weight predicts more disordered eating behaviours and body dissatisfaction than greater weight. This study highlighted the potential role of being teased for developing disordered eating and body dissatisfaction in thinner persons. Additionally, it emphasises the implications of weight-teasing for fatter individuals during adolescence and adulthood.

In general, findings from the present study are consistent with the results of cross-sectional studies using different samples and measurements. For instance, in a study of 347 undergraduate adults, being teased about their weight and BMI were significantly correlated with body dissatisfaction variables (weight concern, shape concern and appearance, self-esteem) in male and female samples [16]. Moreover, in adolescent samples, a positive relationship was found in concurrent and longitudinal research [31,32,33]. In addition, the positive relations found in our results between weight-teasing and body dissatisfaction, disordered eating behaviours and weight-control behaviours were also reported in a meta-analysis by Menzel et al. [7]. In this study, Menzel found a medium-large effect for the relationship between weight-teasing and body dissatisfaction, moderate associations between weight-teasing and disordered eating, weight-teasing and bulimic behaviours, and also between weight-teasing and dietary restraint. 

Within the present paper, we introduced the results of adolescents and adults. Although the study is not longitudinal, our results align with those of Puhl et al. [34], who examined adolescents longitudinally for 15 years. They found that weight-teasing in adolescence predicted body dissatisfaction and obesity in adulthood. In addition, Neumark-Sztainer et al. [35] found cross-sectional and longitudinal associations between weight-teasing, weight and body dissatisfaction, and weight-control behaviours, among others. Likewise, we found that weight-teasing predicted body dissatisfaction, disordered eating, and weight outcomes in adolescents and adults. In summary, our results showed that weight-teasing is related to weight outcomes in adolescence and adulthood.

Concerning gender, the literature indicates that the prevalence of disordered eating, body dissatisfaction and weight-teasing are higher among females than among males (e.g., [35]). In the same line, our study showed that adolescent girls scored slightly higher in being teased about weight than boys, which coincides with the findings of Rojo-Moreno et al. [36] in a study of 57,997 Spanish adolescents. Epidemiological studies have reported that females experience more weight-teasing than males in general and are more affected by weight-teasing than males [2,37]. Nevertheless, a meta-analysis by Emmer et al. [3] found gender as a non-significant result in the relationship between weight-teasing and eating disorders (N = 59,177). Moreover, our results in the adult sample showed no differences in weight-teasing, suggesting that, among adults, the experience of being teased about one’s weight may be egalitarian between women and men. In this line, our results are also supported by Benas et al. [38], who found no gender moderation in weight-teasing, disordered eating, and body dissatisfaction in a sample of undergraduates. An explanation proposed by Emmer et al. [3] is centred on the ideal of beauty as key to the feminine self-concept in adolescence, where physical appearance and body shape-size receive increased attention. On the contrary, adult identity is not so much conditioned by physical appearance, as other emotional and psychological factors are more significant in this stage [39].

Regarding the consistent weight-teasing effects on disordered eating and body dissatisfaction after controlling for weight in adults and adolescents in the present paper, our findings denote the importance of both predictors in developing lifetime body image disturbance and disordered eating behaviours, as other research also suggests [36]. Beyond these findings, Thompson et al. [14] in a longitudinal study, predicted the onset of dissatisfaction, restrictive eating behaviours and bulimia from different levels of weight and weight-teasing, suggesting an interactive influence of the development of body image disturbances.

Thus, in the examination of weight as a potential moderator of weight-teasing, body dissatisfaction and disordered eating behaviours, our interaction effects—albeit small—are close to the range described by McClelland [40], where interactions usually account for 1% to 3% of the variance above and beyond the main effects. The significant interaction effects of our study imply that if the levels of weight-teasing received are low, then greater weight will predict higher levels of disordered eating behaviours and body dissatisfaction. However, if the level of weight-teasing received is high, our results showed that lower weight predicts more disordered eating behaviours and body dissatisfaction than greater weight. Some research has suggested that greater weight has been positively associated with high weight-teasing experience, and lower weight with low teasing experience [41]. However, not all the literature coincides with this view. Kohlmann et al. [42] showed a significant but small association between weight status and body image. In addition, the correlation increased when they considered weight-teasing as a mediator variable. These authors concluded that it is highly important to consider underweight and overweight status in the relationship between weight-teasing and body image. In fact, our interaction results add that thinner people who are targets of weight-teasing may also develop disordered eating and body dissatisfaction, depending on the weight-teasing levels received. 

Furthermore, the controversy remains regarding body dissatisfaction. The fact that the interaction was non-significant in only one questionnaire used to measure body dissatisfaction in adolescents is noteworthy. Additionally, other research found different results in body dissatisfaction measured by BSQ and EDI-2-BD [43]. A potential explanation for this result is found in the conceptualisation of the questionnaires: BSQ assesses mainly emotional, cognitive, perceptual, and behavioural aspects, whereas EDI measures primarily affective and cognitive characteristics. Regarding the adult sample, previous research linked weight-teasing and weight-control behaviours in adult samples (e.g., [44]). Similarly, our results showed that they are related and that weight-teasing affects weight-control behaviours, although the resulting interaction term was non-significant. Accordingly, Puhl et al. [34] found a relationship between weight-teasing and weight-control behaviours, but weight-teasing did not predict weight-control behaviours in an adult sample. Conversely, weight-teasing longitudinally predicted other weight outcomes such as body dissatisfaction, BMI, and disordered eating. Conjointly, weight-teasing did not explain weight-control behaviours during adulthood. Additionally, in a study where weight-teasing was strongly linked to unhealthy weight-control behaviours, the association was measured only for a short period of time. This seems to suggest that weight-teasing may have no effects over a long period on weight-control behaviours [45]. Further research should address these aspects to evaluate their relevance in disordered eating behaviours.

### Limitations

The current study has several strengths: mainly, its focus on weight as a moderator in the relationship between weight-teasing and body dissatisfaction, disordered eating and weight-control behaviours. Another strong point is the data analysis with two sufficiently large samples, adolescents and adults, and the different instruments employed. At the same time, the study has some limitations that must be considered when interpreting the findings. Firstly, the cross-sectional nature of the study does not allow causal inferences. Future studies should investigate the detected relations longitudinally. Secondly, our measures of weight-teasing did not record the origin of these negative comments or the period in the adult sample when the weight-teasing occurred. Peer teasing has been related to low self-esteem, high depressive symptoms [10], unhealthy weight-control practices and binge eating [10]. Furthermore, weight-related comments from a romantic partner were not associated with adults’ self-esteem or intentions to lose weight, but weight-related remarks from family members were linked to disordered eating behaviours and psychosocial problems [46]. Altogether, this suggests that the origin and the period (adulthood or adolescence) of the teasing play a role in the different eating disorders, attitudes and behaviours. Finally, the current research is a first step, but alternative measures may be needed to assess weight-teasing and body image variables among a broad weight-range population, also considering thin people. 

## 5. Conclusions

This study contributes to the actual knowledge on this subject by highlighting the potential role of thinner persons being teased in the development of disordered eating and body dissatisfaction. Additionally, it emphasises the implications of weight-teasing for fatter individuals in adolescence and adulthood. The results provide sufficient impetus for continued consideration of the complexity of the relationship between weight-teasing and body image over the lifespan.

## Figures and Tables

**Table 1:** Descriptive statistics and correlations among the variables of the study in the adolescent sample.

Variables	1	2	3	4	5	6	7	8
1. BSQ	-	**0.72**	**0.54**	**0.62**	**0.29**	**0.14**	**−0.02**	**−0.23**
2. EDI-2 DT		-	**0.75**	**0.75**	**0.37**	**0.29**	**−0.02**	**−0.28**
3. EDI-2 BD			-	**0.69**	**0.40**	**0.33**	**0.03**	**−0.23**
4. ChEAT				-	**0.44**	**0.32**	**0.07**	**−0.37**
5. POTS-WT					-	**0.38**	−0.01	**−0.04**
6. R-Weight						-	0.00	0.00
7. Age							-	0.02
8. Gender								-
*M*	8.39	3.50	6.33	61.44	9.09	0.00	15.25	-
*SD*	7.29	4.62	5.12	32.60	8.15	10.59	1.20	-

Note: Significant correlations are shown in bold. *p* < 0.05.

**Table 2:** Hierarchical regression analyses in the adolescent sample.

	BSQ				EDI-2 DT				EDI-2 BD				ChEAT			
	Step 1		Step 2		Step 1		Step 2		Step 1		Step 2		Step 1		Step 2	
Predictors	β	*p*	β	*p*	β	*p*	β	*p*	β	*p*	β	*p*	β	*p*	β	*p*
Gender	−0.35	<0.001	−0.35	<0.001	−0.27	<0.001	−0.27	<0.001	−0.22	<0.001	−0.22	<0.001	−0.22	<0.001	−0.22	<0.001
Age	0.07	<0.001	0.07	<0.001	−0.02	0.020	−0.02	0.023	0.03	<0.001	0.03	<0.001	−0.01	0.20	−0.01	0.217
Residual weight	0.18	<0.001	0.24	<0.001	0.18	<0.001	0.23	<0.001	0.21	<0.001	0.21	<0.001	0.03	<0.001	0.08	<0.001
POTS-WT	0.36	<0.001	0.41	<0.001	0.29	<0.001	0.32	<0.001	0.32	<0.001	0.32	<0.001	0.27	<0.001	0.30	<0.001
Residual Weight × POTS-WT	-	-	−0.11	<0.001	-	-	−0.08	<0.001	-	-	−0.01	0.77	-	-	−0.08	<0.001
Δ*R*^2^	0.36		0.003		0.23		0.002		0.25		0.00		0.13		0.002	

Note: *p* < 0.05.

**Table 3:** Descriptive statistics and correlations among the variables of the study in the adult sample.

Variables	1	2	3	4	5	6	7
1. WCB	-	**0.72**	**0.52**	**0.37**	**0.23**	**−0.16**	**−0.17**
2. EDI-DT		-	**0.73**	**0.39**	**0.24**	**−0.15**	**−0.36**
3. EDI-BD			-	**0.43**	**0.35**	**−0.20**	**−0.34**
4. POTS-WT				-	**0.40**	−0.09	−0.02
5. BMI					-	**0.30**	**0.15**
6. Age						-	0.07
7. Gender							-
*M*	4.90	20.34	30.98	13.49	23.16	29.98	-
*SD*	3.21	8.39	11.47	13.34	3.78	11.00	-

Note: Significant correlations are shown in bold. *p* < 0.05.

**Table 4:** Hierarchical regression analyses in the adult sample.

	WCB				EDI-DT				EDI-BD			
	Step1		Step 2		Step 1		Step 2		Step 1		Step 2	
Predictors	β	*p*	β	*p*	β	*p*	β	*p*	β	*p*	β	*p*
Gender	−0.18	<0.001	0.18	<0.001	0.38	<0.001	−0.38	<0.001	0.37	<0.001	0.37	<0.001
Age	−0.19	<0.001	0.19	<0.001	0.18	<0.001	−0.19	<0.001	0.27	<0.001	0.27	<0.001
BMI	0.21	0.001	0.26	0.002	0.25	<0.001	0.44	<0.001	0.39	<0.001	0.50	<0.001
POTS-S-WT	0.26	<0.001	0.52	0.064	0.26	<0.001	1.16	<0.001	0.24	<0.001	0.77	0.002
BMI × POTS-S-WT	-	-	0.29	0.351	-	-	–1.00	<0.001	-	-	0.59	0.025
Δ*R*^2^	0.21		0.002		0.36		0.027		0.42		009	

Note: *p* < 0.05.

## Data Availability

Datasets analysed or generated during the study are under the control of the investigators at the correspondence lau@unizar.es.
